# Parental Midlife Body Shape and Association with Multiple Adult Offspring Obesity Measures: North West Adelaide Health Study

**DOI:** 10.1371/journal.pone.0137534

**Published:** 2015-09-10

**Authors:** Janet F. Grant, Catherine R. Chittleborough, Anne W. Taylor

**Affiliations:** 1 Population Research and Outcome Studies, Discipline of Medicine, The University of Adelaide, Adelaide, South Australia, Australia; 2 School of Public Health, The University of Adelaide, Adelaide, South Australia, Australia; University of Alabama at Birmingham, UNITED STATES

## Abstract

There is compelling evidence that parental weight is a strong determinant of offspring weight status. The study used cross-sectional self-reported and measured data from a longitudinal cohort of Australian adults (n = 2128) from Stage 3 (2008–10) of the North West Adelaide Health Study (1999–2003, baseline n = 4056) to investigate the association between midlife parental body shape and four indicators of obesity and fat distribution. The analysis used measured body mass index (BMI), waist circumference (WC), waist hip ratio (WHR) and waist height ratio (WHtR) of adult offspring, together with pictograms for recall of parental body shape. Compared to both parents being a healthy weight, offspring were more likely to be overweight or obese if both parents were an unhealthy weight at age 40 (OR 2.14, 95% CI 1.67–2.76) and further, those participants whose mother was an unhealthy weight were more likely to be overweight or obese themselves (OR 1.50, 95% CI 1.14–1.98). There were similar but lower results for those with an overweight/obese father (OR 1.44, 95% CI 1.08–1.93). The effect of one or both parents being overweight or obese tended to be stronger for daughters than for sons across BMI, WC and WHtR. BMI showed the strongest association with parental body shape (OR 2.14), followed by WC (OR 1.78), WHtR (OR 1.71) and WHR (OR 1.45). WHtR (42–45%) and BMI (35–36%) provided the highest positive predictive values for overweight/obesity from parental body shape. Parental obesity increases the risk of obesity for adult offspring, both for overall body shape and central adiposity, particularly for daughters. Pictograms could potentially be used as a screening tool in primary care settings to promote healthy weight among young adults.

## Introduction

Research suggests that the location of excess body fat within individuals is associated with morbidity and mortality [1]. Furthermore, cardiometabolic complications are more likely to occur when visceral fat storage is present in excess [2]. Obesity is the most recent major global epidemic, rarely appearing as a health issue before the 20th century but doubling in rate since 1980 [3]: it is also a major problem in Australia with 35.3% of the population being overweight and 27.5% being obese in 2011–12.[4]

Accurate assessment of body fat distribution on a large-scale population basis can be problematic due to increased costs and portability of valid medical technologies. Population-level proxy measures can therefore be used to determine health risk through the categorisation of obesity [5] by indices such as body mass index (BMI) and central adiposity measures including waist circumference (WC), waist hip ratio (WHR) [6] and waist height ratio (WHtR) [7]. Existing literature propone pictograms, representing body size and shape, as a valid approach to estimating personal BMI [8, 9], and recalling parental weight [10].

There is compelling evidence that parental weight is a strong determinant of offspring weight status [11–13]. A 2012 study of three generations examined the relative maternal and paternal associations and reported an enduring association between mother and offspring BMI [14]. Recent research has explored the relative influence of both maternal and paternal factors such as parental smoking, poor diet, low rates of physical activity and lower social class, together with mother’s older age and weight gain during pregnancy, may negatively impact on offspring health [11, 15, 16]. Findings from another recent study support the conclusion that maternal BMI has a significantly stronger influence on adult female offspring BMI despite the fact that both parents' BMI influence adult male offspring BMI equally [17].

Currently, available data relating to the association between parental body shape and adult offspring weight status predominantly use BMI. Fewer studies incorporate measures of central adiposity.

This study aimed to assess if there was an association between midlife parental body shape and four measures of obesity and fat distribution among Australian adults. Combining an indication of parental body shape as a screening device, together with a person’s current body shape measure, may be useful in primary care to assist in the early identification of those who may be at an increased risk of developing obesity and related co-morbidities, for targeting purposes for regular monitoring, intervention and treatment.

## Methods

### Sample

The North West Adelaide Health Study (NWAHS) is a representative longitudinal study of 4056 randomly selected adults aged 18 years and over, recruited from 1999 to 2003 from the north-west region of Adelaide, the capital of South Australia. Participants were recruited using the Electronic White Pages and during the initial Computer Assisted Telephone Interview (CATI), the eligible adult who had the most recent birthday in the household was invited to participate. People were excluded if they did not have the capacity to participate due to illness or intellectual limitations, if they were unable to communicate in English or if they lived in a residential institution. The study methodology has previously been described in detail [18, 19]. Written informed consent was gained from study participants. Ethical approval for this research was granted by the Human Research Ethics Committee of The University of Adelaide.

NWAHS participants have been followed up several times since initial recruitment. Measured anthropometric data used in this paper are from Stage 1 (baseline 1999–2003, response rate 49.1%) and Stage 3 (second follow-up 2008–2010, overall n = 2871 (questionnaire n = 2483, clinic n = 2487)), response rate 76.0%). Self-reported information was also collected by CATI and self-completed questionnaire at both stages, as well as via a telephone follow up survey in 2007 (TFU2, n = 2996, response rate 90.2%).

Participants who attended all three major stages of the study and who provided information about their parents' occupation and country of birth in TFU2, as well as their parents' body shape in the Stage 3 questionnaire, were included in the study. This reduced the overall sample from 4056 to 2128, after excluding those without biomedical information at each major stage or related information about at least one of their parents. There were 176 participants who provided information on only parent (mother only n = 119; father only n = 57), resulting in a multinomial regression analysis sample of 1952 who provided body shape information on both parents.

### Offspring body shape

Four anthropometric measures of adult offspring were undertaken. Height without shoes was measured to the nearest 0.5 centimetres using a wall-mounted stadiometer (height measurement), and weight to the nearest 0.1 kilogram in light clothing and without shoes using standard digital scales. BMI was calculated by dividing the participant's weight in kilograms by the square of their height in metres (kg/m^2^). BMI values were initially grouped according to the World Health Organization BMI classifications [5] and then reduced to three categories for analysis: underweight/healthy weight (BMI <25), overweight (BMI 25–29) and obese (BMI ≥30).

Waist circumference (WC) was measured to the nearest 0.1 centimetre using an inelastic tape maintained in a horizontal plane, with the subject standing comfortably with weight distributed evenly on both feet. The measurement was taken at the level of the narrowest part of the waist. Hip circumference was also measured using an inelastic tape, at the level of the maximum posterior extension of the buttocks. Three measurements of the waist and hip were taken and the mean for each was calculated. The cut-off points for recommended weight reduction to reduce major cardiovascular risk factors using WC were ≥102 cm for men and ≥88 cm for women [20], and a waist-hip ratio (WHR) of >1.0 for men and >0.85 for women [21]. The cut-off points for waist-height ratio (WHtR) for a reduction in cardiometabolic outcomes was 0.5 [22].

After their clinic examination, participants were provided with selected results with an indication of where these results were outside desirable levels (including BMI <18.5 or >24.9, blood pressure >140/90mmHg, total cholesterol >7.0mmol/L, glucose >7.0mmol/L and lung function >80% predicted for age and sex of forced expiratory volume in one second (FEV1)), while their general practitioner was provided with all results, including blood and urine pathology, blood pressure and lung function, BMI and WHR.

### Parental body shape

**Fig 1:**
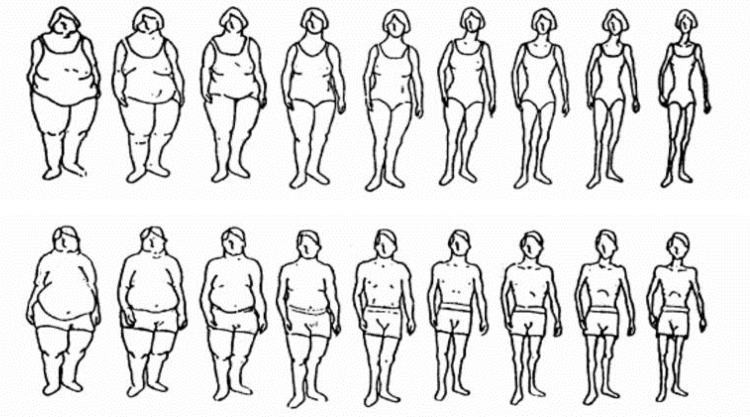
Images for perceived body shape of parents from the Figure Rating Scale (adapted from the paper by Sorensen et al [8]) used in the North West Adelaide Health Study. Silhouettes 1–2 = Very overweight; Silhouettes 3–4 = Moderately overweight; Silhouette 5 = Slightly overweight; Silhouettes 6–7 = Appropriate (healthy) weight; Silhouettes 8–9 = Underweight. Reprinted with permission.

Parental body shape was asked in the Stage 3 self-completed questionnaire, and operationalized through the use of a set of nine figures from a set of validated pictograms. The pictograms ask respondents to identify the body type of their biological mother and father at age 40 (Fig 1). For analysis purposes, the set of figures were each derived into a dichotomous variable for mothers and fathers: figures 1 through 5 were classified as unhealthy weight, and figures 6 through 9 were classified as healthy weight/underweight [23].

### Demographics

Demographic variables at both Stage 1 (baseline) and Stage 3 included marital status, work status, highest level of education achieved and gross annual household income. Household tenure was asked only in Stage 3. Country of birth was asked at baseline for participants and in TFU2 for their parents. Occupation data regarding participants and their parents was asked in TFU2 and was coded into eight major groups based on the Australian and New Zealand Standard Classification of Occupations [24].

### Statistical analysis

The data were analysed using SPSS Version 20.0 (IBM, Armonk, NY). Univariable analyses using chi-square tests compared demographic and body shape proportions of daughters and sons at baseline and at follow-up, as well as the reported body shape of their parents at midlife at follow-up. Baseline anthropometric measures were used in the univariate analyses to reduce the effect of possible bias from participation in a longitudinal study and action from feedback of clinical information.

Parental body shape via pictograms was used in the absence of measurements. The silhouettes were further classified into four categories for use as the independent variable: both parents healthy weight, only father unhealthy weight, only mother unhealthy weight and both parents unhealthy weight. Statistical analysis regarding the association of offspring body shape with parental body shape was cross-sectional. Using both parents having a healthy weight as the reference category, unadjusted odds ratios (together with proportions, 95% confidence intervals and p values) were calculated across the four weight measures on those participants who had provided information about parental body shape for both parents (n = 1952). Sensitivity (true positives), specificity (true negatives), and positive and negative predictive values of parental body shape forecasting offspring obesity were calculated for those adult offspring who had a measured BMI <25, using dichotomous variables for both the recognised cut-offs of each weight measure and the pictogram silhouettes.

## Results

### Demographics

Table 1 provides an overview of selected demographic and life-course variables for participants and their parents from baseline and/or follow-up (Stage 3 or TFU2 where indicated). A comparison of selected demographic variables between baseline and the analysis sample is show in S1 Table.

**Table 1:** Socio-demographics for study participants for baseline and follow up.

		DAUGHTERS				SONS			
*(self reported)*		Baseline		Follow Up		Baseline		Follow Up	
SOCIO-DEMOGRAPHICS		*n*	%	*n*	%	*n*	%	*n*	%
**Age**	18 to 29 years	*84*	7.5	*17*	1.5	*87*	8.7	*24*	2.4
	30 to 39 year	*183*	16.3	*91*	8.1	*164*	16.4	*86*	8.6
	40 to 49 years	*300*	26.6	*223*	19.8	*239*	23.9	*199*	19.9
	50 to 59 years	*275*	24.4	*294*	26.1	*249*	24.9	*230*	23.0
	60 to 69 years	*186*	16.5	*258*	22.9	*165*	16.5	*249*	24.9
	70 years and over	*98*	8.7	*243*	21.6	*98*	9.8	*214*	21.4
**Marital status**	Married/defacto	*737*	65.5	*719*	63.8	*685*	68.4	*714*	71.3
	Separated/divorced	*158*	14.0	*156*	13.9	*135*	13.5	*123*	12.3
	Widowed	*116*	10.3	*157*	13.9	*40*	4.0	*59*	5.9
	Never married	*110*	9.8	*80*	7.1	*140*	14.0	*85*	8.5
	Not stated	*5*	0.4	*14*	1.3	*2*	0.2	*21*	2.1
**Work status**	Full time employed	*294*	26.1	*297*	26.4	*599*	59.8	*523*	52.2
	Part time / casual employment	*307*	27.3	*265*	23.5	*83*	8.3	*74*	7.4
	Unemployed	*24*	2.1	*18*	1.6	*29*	2.9	*16*	1.6
	Home duties	*264*	23.4	*67*	6.0	*8*	0.8	*3*	0.3
	Retired	*180*	16.0	*393*	34.9	*228*	22.8	*326*	32.5
	Student	*18*	1.6	*6*	0.5	*22*	2.2	*4*	0.4
	Other	*25*	2.2	*65*	5.8	*29*	2.9	*34*	3.4
	Not stated	*14*	1.2	*15*	1.3	*4*	0.4	*22*	2.2
**Highest educational qualification**	Up to & incl secondary	*677*	60.1	*624*	55.4	*358*	35.7	*307*	30.6
	Trade/Apprenticeship/Certificate/ Diploma	*276*	24.5	*250*	22.2	*503*	50.2	*467*	46.6
	Bachelor degree or higher	*160*	14.2	*239*	21.2	*134*	13.4	*207*	20.7
	Other/Don’t know/Not stated	*13*	1.2	*13*	1.2	*7*	0.7	*21*	2.1
**Income (gross annual household)**	Up to $12,000	*147*	13.1	*30*	2.7	*58*	5.8	*16*	1.6
	$12,001–$20,000	*174*	15.5	*157*	13.9	*104*	10.4	*103*	10.3
	$20,001 to $40,000	*263*	23.4	*280*	24.9	*295*	29.4	*213*	21.3
	$40,001 to $60,000	*251*	22.3	*157*	13.9	*252*	25.1	*178*	17.8
	$60,001 to $80,000	*131*	11.6	*133*	11.8	*120*	12.0	*141*	14.1
	More than $80,000	*111*	9.9	*251*	22.3	*141*	14.1	*290*	28.9
	Not stated	*49*	4.4	*118*	10.5	*32*	3.2	*61*	6.1
**Country of birth**	Australia	*785*	69.7			*701*	70.0		
	United Kingdom/Ireland	*217*	19.3			*172*	17.2		
	Europe	*86*	7.6			*95*	9.5		
	Asia/ Oceania/ Americas/ Africa	*31*	2.8			*33*	3.3		
	Other/Not stated	*7*	0.6			*1*	0.1		
* **Occupation**	Manager	*27*	2.4			*67*	6.7		
	Professional	*190*	16.9			*144*	14.4		
	Technician or trade worker	*58*	5.2			*289*	28.8		
	Community or personal service worker	***90***	8.0			*37*	3.7		
	Clerical or admin worker	***320***	28.4			*145*	14.5		
	Sales worker	*122*	10.8			*67*	6.7		
	Machinery operator or driver	*28*	2.5			*87*	8.7		
	Labourer	*98*	8.7			*132*	13.2		
	Unable to classify, economically inactive or not stated	*193*	17.1			*34*	3.4		
**Housing tenure**	Owned or being purchased by the occupants			959	85.2			858	85.6
	Renting/board			127	11.3			98	9.8
	A retirement village/unit, nursing home, life tenure			24	2.1			21	2.1
	Other/Not stated			16	1.4			25	2.5
* **Mother's country of birth**	Australia			*667*	59.2			*569*	56.8
	United Kingdom/Ireland			*263*	23.4			*225*	22.5
	Europe			*164*	14.6			*169*	16.9
	Asia/Oceania/Americas/Africa			*32*	2.8			*38*	3.8
	Not stated			-	-			*1*	0.1
* **Father's country of birth**	Australia			*612*	54.4			*550*	54.9
	United Kingdom/Ireland			*273*	24.2			*221*	22.1
	Europe			*188*	16.7			*187*	18.7
	Asia/Oceania/Americas/Africa			*42*	3.7			*34*	3.4
	Not stated			*11*	1.0			*10*	1.0
* **Mother's occupation**	Manager			***57***	**5.1**			*34*	3.4
	Professional			*81*	7.2			*65*	6.5
	Technician or trade worker			*71*	6.3			*49*	4.9
	Community or personal service worker			*56*	5.0			*52*	5.2
	Clerical or admin worker			***89***	7.9			*60*	6.0
	Sales worker			*67*	6.0			*59*	5.9
	Machinery operator or driver			*2*	0.2			*5*	0.5
	Labourer			*138*	12.3			*108*	10.8
	Unable to classify, economically inactive or not stated			*565*	50.2			***570***	**56.9**
* **Father's occupation**	Manager			*166*	14.7			*132*	13.2
	Professional			*108*	9.6			*105*	10.5
	Technician or trade worker			*253*	22.5			*245*	24.5
	Community or personal service worker			*53*	4.7			*41*	4.1
	Clerical or admin worker			*85*	7.5			*78*	7.8
	Sales worker			*56*	5.0			*62*	6.2
	Machinery operator or driver			*105*	9.3			*72*	7.2
	Labourer			*270*	24.0			*247*	24.7
	Unable to classify, economically inactive or not stated			*30*	2.7			*20*	2.0
**TOTAL**		**1126**	**100.0**	**1126**	**100.0**	**1002**	**100.0**	**1002**	**100.0**

*Asked in the Telephone Follow Up survey, 2007.

### Body shape of offspring and parents

Table 2 examines the proportion of female and male offspring participants within each category of four measures of body shape at baseline and second follow-up, with parental weight status.

**Table 2:** Body shape of study participants for baseline and second follow up, and body shape of the parent(s).

		DAUGHTERS				SONS			
		Baseline		Follow-up		Baseline		Follow-up	
		*n*	%	*n*	%	*n*	%	*n*	%
**OFFSPRING *(measured)***									
**BMI**	Underweight/Healthy weight (<25)	*429*	38.1	*349*	31.0	*241*	24.1	*191*	19.1
	Overweight (25–29)	*367*	32.6	*387*	34.4	*493*	49.2	*477*	47.6
	Obese (≥30)	*330*	29.3	*390*	34.6	*268*	26.7	*333*	33.2
**Central adiposity**	Android obesity (WHR>1.0 males; >0.85 females)	*284*	25.2	*430*	38.2	*110*	11.0	*246*	24.6
	High WC (≥102cm males; ≥88cm females)	*469*	*41*.*7*	*571*	*50*.*7*	*367*	*36*.*6*	*460*	*45*.*9*
**High WHtR** (≥0.5)		*686*	60.9	*777*	69.0	*824*	82.2	*853*	85.1
**PARENTS’ BODY SHAPE AT MID-LIFE (*pictograms*)**									
**Mother**	Underweight/ Healthy weight			*444*	39.4			*406*	40.5
	Overweight			*614*	54.5			*537*	53.6
	Obese			*41*	3.6			*29*	2.9
	Not stated			*27*	2.4			*30*	3.0
**Father**	Underweight/ Healthy weight			*492*	43.7			*419*	41.8
	Overweight			*535*	47.5			*521*	52.0
	Obese			*26*	2.3			*16*	1.6
	Not stated			*73*	6.5			*46*	4.6
**TOTAL**		**1126**	**100.0**	**1126**	**100.0**	**1002**	**100.0**	**1002**	**100.0**

Overall, using World Health Organization BMI classifications at baseline (unweighted data), 0.8% (n = 17) of the 2128 participants were underweight (BMI <20); 30.7% (n = 653) were normal weight (BMI 20–24); 40.4% (n = 860) were overweight (BMI 25–29); and 28.1% (n = 598) were obese. Of those who were obese, 65.9% (n = 394) were in Obese Class I (BMI 30–34), 25.6% (n = 153) were in Obese Class II (BMI 35.00 to 39.99) and 8.5% (n = 51) were in Obese Class III (BMI ≥40), with daughters more likely than sons to be in the latter (heavier) two obese classes (not shown).

Regarding central adiposity overall, 18.5% of participants had a high waist hip ratio (WHR men >1.0; women > 0.85); 39.3% had a high waist circumference (WC men ≥102 cm; women ≥88 cm) and 71.0% had a high waist height ratio (WHtR ≥0.5).

The BMI of study participants increased from a mean of 27.80 (SD 5.21) at Stage 1 over approximately seven years to 28.66 (SD 5.48) at Stage 3, with a corresponding increase in the mean waist circumference from 92.23 cm (SD 14.31) to 95.0 cm (SD 14.97) (not shown). Overall, 1322 participants (62.1%) gained weight (mean 6.0 kg, 95% CI 5.67–6.30) between Stage 1 and Stage 3. Of those, daughters gained slightly more weight (n = 699, mean 6.1 kg, 95% CI 5.73–6.55, range 0.05 to 34.0 kg) than sons (n = 623, mean 5.8 kg, 95% CI 5.34–6.29, range 0.05 to 60.7 kg). Those participants who gained weight were more likely to be younger (aged 18 to 49 years) and male.

There were also 798 participants (37.5%) who lost weight (mean 4.7 kg, 95% CI 4.36–5.10%) during the same timeframe. Of those, more daughters lost slightly more weight (n = 421, mean 5.2kg, 95% CI 4.61–5.71, range 0.05 to 46.0 kg) than sons (n = 377, mean 4.3kg, 95% CI 3.76–4.74, range 0.05 to 41.2 kg).

There were no differences between daughters and sons regarding their responses to the question about their parents’ body shape at midlife.

Table 3 provides a comparison of the four measures of obesity and central adiposity, with four combinations of parental overall body shape, as well as for daughters and sons. Regardless of which body shape measure was used, there was strong evidence that offspring were more likely to be overweight or obese if both parents were an unhealthy weight at age 40 when compared to those whose parents were a healthy weight. For example, using BMI and the reference category as both parents being a healthy weight, the overall odds ratio (OR) for BMI when both parents have an unhealthy weight was 2.14 (95% CI 1.67–2.76). There was moderate evidence that an unhealthy maternal body shape influenced their offspring’s adult body shape when compared to both parents being a healthy weight (OR 1.50, 95% CI 1.14–1.98), with a slightly lower result for unhealthy paternal body shape (OR 1.44, 95% CI 1.08–1.93). The effect of one or both parents being overweight or obese tended to be stronger for daughters than for sons regardless of whether one or both parents were an unhealthy weight for BMI, WC and WHtR (e.g. BMI daughters/sons—OR both parents 2.36, 1.92; mother only 1.87, 1.17; father only 1.54; 1.28 respectively). BMI showed the strongest association with parental body shape (OR 2.14), followed by WC (OR 1.78), WHtR (OR 1.71) and WHR (OR 1.45).

**Table 3:** Unadjusted odds ratios (proportions, 95% confidence intervals and p values) for overweight/obese offspring measures of parental body shape/weight.

Overweight/obese (Stage 1)*		Both parents healthy weight	Father UNHEALTHY weight				Mother UNHEALTHY weight				Both parents UNHEALTHY weight			
		Overall n = 453	Overall n = 353				Overall n = 431				Overall n = 715			
		*Daughters n = 242*	*Daughters n = 176*				*Daughters n = 239*				*Daughters n = 369*			
		*Sons n = 211*	*Sons n = 177*				*Sons n = 192*				*Sons n = 346*			
		Ref 1.0												
(measured)		n (%)	n (%)	OR	95% CI	p value	n (%)	OR	95% CI	p value	n (%)	OR	95% CI	p value
**BMI overall**		**264 (58.3%)**	**236 (66.9%)**	**1.44**	**(1.08–1.93)**	**0.013**	**292 (67.7%)**	**1.50**	**(1.14–1.98)**	**0.004**	**536 (75.0%)**	**2.14**	**(1.67–2.76)**	**<0.001**
	Daughters	117 (48.3%)	104 (59.1%)	1.54	(1.04–2.28)	0.030	152 (63.6%)	1.87	(1.30–2.69)	0.001	254 (68.8%)	2.36	(1.69–3.30)	<0.001
	Sons	147 (69.7%)	132 (74.6%)	1.28	(0.82–2.00)	0.284	140 (72.9%)	1.17	(0.76–1.81)	0.472	282 (81.5%)	1.92	(1.29–2.86)	0.001
**WC overall**		**140 (30.9%)**	**127 (36.0%)**	**1.26**	**(0.94–1.69)**	**0.129**	**176 (40.8%)**	**1.54**	**(1.17–2.04)**	**0.002**	**317 (44.3%)**	**1.78**	**(1.39–2.28)**	**<0.001**
	Daughters	74 (30.6%)	69 (39.2%)	1.46	(0.97–2.20)	0.067	99 (41.4%)	1.61	(1.10–2.34)	0.013	175 (47.4%)	2.05	(1.46–2.88)	<0.001
	Sons	66 (31.3%)	58 (32.9%)	1.07	(0.70–1.64)	0.754	77 (40.1%)	1.47	(0.98–2.22)	0.065	142 (41.0%)	1.53	(1.07–2.19)	0.021
**WHtR overall**		**292 (64.5%)**	**236 (66.9%)**	**1.11**	**(0.83–1.49)**	**0.478**	**303 (70.3%)**	**1.31**	**(0.98–1.73)**	**0.064**	**541 (75.7%)**	**1.71**	**(1.33–2.22)**	**<0.001**
	Daughters	126 (52.1%)	104 (59.1%)	1.33	(0.90–1.97)	0.154	140 (58.6%)	1.30	(0.91–1.97)	0.151	245 (66.4%)	1.82	(1.31–2.54)	<0.001
	Sons	166 (78.7%)	132 (74.6%)	0.80	(0.50–1.28)	0.341	163 (84.9%)	1.52	(0.91–1.28)	0.109	296 (85.5%)	1.60	(1.03–2.51)	0.037
**WHR overall**		**69 (15.2%)**	**54 (15.3%)**	**1.01**	**(0.68–1.48)**	**0.979**	**84 (19.5%)**	**1.35**	**(0.95–1.91)**	**0.095**	**148 (20.7%)**	**1.45**	**(1.06–1.99)**	**0.020**
	Daughters	50 (20.7%)	44 (25.0%)	1.28	(0.81–2.03)	0.295	60 (25.1%)	1.29	(0.84–1.97)	0.247	101 (27.4%)	1.45	(0.98–2.13)	0.061
	Sons	19 (9.0%)	10 (5.6%)	0.61	(0.27–1.34)	0.215	24 (12.5%)	1.44	(0.76–2.73)	0.258	47 (13.6%)	1.59	(0.90–2.79)	0.107

Note: n = 1952 (176 participants provided parental body shape about only one parent).

* Defined as: BMI > 25; high WHR (1.00 males, 0.85 females); high WC (≥102cm males, ≥88cm females); high WHtR >0.05).

Table 4 shows the sensitivity, specificity, positive and negative predictive values of parental body shape predicting offspring obesity, for those participants who were underweight or normal weight as measured by BMI at baseline (n = 670; male 241, female 429), using four measures of weight status at Stage 3. The highest positive predictive values (PPV) were for both WHtR (overall mothers-fathers 41.8–45.1%; daughters 35.4–36.0%; sons 51.9–62.0% respectively) and BMI (overall mothers-fathers 35.4–36.4%; daughters 31.1–33.5; sons 41.9–42.3% respectively). Sensitivity of parental overweight/obesity in pictograms in predicting overweight/obesity in offspring ranged from 45.2% to 61.3% across all four offspring body shape measures.

**Table 4:** Sensitivity, specificity, and positive and negative predictive values of weight measures based on parental overweight/obesity status for previously underweight or normal weight adult offspring.

Weight measures above cut-offs by gender and parental weight status		Sensitivity	Specificity	Positive Predictive Value	Negative Predictive Value
**WHtR**					
Daughters	Mothers	63.3%	46.0%	64.1%	45.0%
	Fathers	56.5%	51.7%	64.2%	43.7%
Sons	Mothers	60.9%	54.1%	86.0%	22.9%
	Fathers	56.3%	44.6%	81.9%	18.6%
**Both**	**Mothers**	**62.0%**	**48.3%**	**74.3%**	**34.5%**
	**Fathers**	**56.4%**	**49.6%**	**72.9%**	**32.2%**
**BMI**					
Daughters	Mothers	65.2%	49.4%	67.3%	47.1%
	Fathers	57.0%	52.7%	65.8%	43.5%
Sons	Mothers	60.6%	49.4%	79.2%	28.3%
	Fathers	58.7%	51.7%	79.0%	28.9%
**Both**	**Mothers**	**62.8%**	**49.4%**	**72.8%**	**38.1%**
	**Fathers**	**57.9%**	**52.3%**	**72.2%**	**36.8%**
**WC**					
Daughters	Mothers	66.2%	45.0%	46.0%	65.3%
	Fathers	58.5%	50.3%	44.9%	63.6%
Sons	Mothers	64.1%	45.2%	40.6%	68.2%
	Fathers	57.8%	44.8%	37.8%	64.7%
**Both**	**Mothers**	**65.2%**	**45.1%**	**43.5%**	**66.7%**
	**Fathers**	**58.2%**	**47.6%**	**41.4%**	**64.1%**
**WHR**					
Daughters	Mothers	65.0%	42.2%	27.2%	78.4%
	Fathers	56.2%	47.7%	26.6%	76.4%
Sons	Mothers	71.0%	43.4%	13.4%	92.4%
	Fathers	56.3%	43.8%	10.8%	89.3%
**Both**	**Mothers**	**66.7%**	**42.8%**	**20.8%**	**85.1%**
	**Fathers**	**56.3%**	**45.7%**	**18.9%**	**82.3%**

## Discussion

This study found that having two obese parents resulted in an increased likelihood of their adult offspring also being overweight or obese. This association tended to be stronger for daughters than sons across BMI, WC and WHtR. Compared to offspring who had both healthy weight parents, those with one parent or both parents who had an unhealthy weight had an increased odds of obesity based on BMI ranging from 44% to 114%. These results were slightly lower based on WC (26 to 78%), WHtR (11 to 71%) and WHR (1 to 45%).

These results support previous findings [11, 25] from predominantly Western societies suggesting that adults with one obese parent during their childhood are more likely to also be obese, with a stronger association if both parents are obese. Overall, when compared with adults who had healthy weight parents, one study observed that adult offspring with obese parents were up to four times more likely to be obese themselves [26].

The proportion of obese South Australians in this study was similar to the national figure (28.1% compared to 26.8%). Our study found that in this population, offspring were more likely to be obese across three of the four measures (BMI, WC and WHtR but not WHR) if their parents were also obese, and the association was stronger for daughters than for sons. Like our study, an earlier study of American families using skinfold thickness measurements reported that mothers of the adult offspring were no more obese than fathers, which may be age-related. In contrast to our study, this study reported no difference in the size of parents of obese sons when compared to obese daughters, which may be due to the different measure used [27]. A study among Canadian families examining obesity risk reported a higher risk ratio for first degree relatives than spouses using BMI, however this was the opposite when using skinfold measurements [25].

Our results also support recent findings from British [11] and Irish [14] studies examining multiple generations suggesting that there is a stronger maternal influence for BMI. The comparable studies used measured data of offspring participants and their children, and reported data for parents. Findings from the British study included that increased maternal BMI was associated with offspring who had a higher consumption of fried foods, a higher level of television watching and smoking, and a lower consumption of fruit. Paternal BMI was considered to have fewer associations with their offspring’s lifestyles in a separate study [28].

There is an ongoing debate regarding the relative contributions of genetic and environmental factors [29, 30]. Repeated early research by one group in Denmark reported a strong association of weight status between adoptees and their biological parents [31]. However, it is argued that the global increase over the past 30 years cannot be explained by biological factors alone and that complex environmental changes, including changes to type and amount of foods consumed, physical activity and socioeconomic factors, play a key role [16, 32].

The majority of earlier studies were based on results from BMI and/or skinfold measurements. A main strength of our study was the ability to compare the association of parental body shape using four clinically measured weight indices. BMI is a composite measure of weight, endorsed by the World Health Organization as the most useful population-level measure [33], as well as being inexpensive and relatively simple to determine by self-report or by clinical measure. WC, WHR and WHtR are indices of abdominal obesity. It is recognised that android or "apple" shaped bodies have a stronger association with obesity-related health risks than gynoid or "pear" shaped bodies [34]. WC alone is useful in predicting this risk [35, 36] and together with BMI, has been shown to have stronger correlations with systolic and diastolic blood pressure than WHR. WC together with hip circumference allows the calculation of waist-hip ratio, providing another measure of centralised fat distribution. WHR is purported to be a more powerful predictor of cardiovascular disease (CVD) related deaths than WC and in turn, more powerful than BMI in both sexes [37]. In a study of adult cardiometabolic risk in different nationalities, WHtR was observed to improve discrimination by 4–5% (compared with BMI) and 3% (compared with WC). WHtR has been shown to be significantly better than WC in screening for diabetes, CVD, hypertension and the metabolic syndrome overall [7, 22]. It is acknowledged that each of these measures have limitations when used in isolation. An examination of BMI, WC and WHR within the NWAHS cohort at baseline was undertaken to explore the limitations of each measure, and to determine if participants would be classified as obese using different criteria. It reported that of those women with a normal BMI, 19.0% had a high WC (≥80 cm), while 8.5% had a high WHR (>0.85). There were corresponding lower proportions for men—3.4% for WC (≥90 cm) and 0.1% for WHR (1.0). Conversely it found that 10.9% of those with a high WHR and 7.8% of those with a high WC were classified as being underweight or normal weight using BMI [38]. Therefore, each measure has a role in identifying people who are overweight or obese with their associated cut-offs being useful as a means to predict risk of chronic disease.

Another strength of the study was the use of clinical rather than self-reported anthropometric measurements, as the latter have been shown to provide an over-estimation of people’s height and an under-estimation of their weight compared to biomedical measures [39].

It was found that identified changes over time in the height-related measures (BMI and WHtR) were not due to any significant variation in participant height. There was minimal loss in height between Stages 1 and 3 mainly due to the effect of age, with the mean height for women being 161.9 cm (SD 6.56) and 161.2 cm (SD 6.74) respectively; and for men 175.5 cm (SD 7.06) and 175.1 cm (SD 7.12) respectively.

Fair to moderate positive predictive values (PPVs) of between 35 to 45% were observed for both WHtR and BMI. This suggests that overall, among those offspring who were underweight or normal weight at baseline and who identified their mother or father as overweight/obese in pictograms, almost half were overweight/obese according to WHtR and one-third were overweight/obese according to BMI at Stage 3. Higher PPVs were seen for sons (52 to 62%) than daughters (~36%). In terms of sensitivity, rates varied from 45% to 61% across all four offspring body shape measures. This suggests that approximately half of overweight/obese offspring could be identified from parental overweight/obese pictograms. The rates of specificity were generally about 52%.

There are limitations in this study that need to be highlighted. These include the use of cross-sectional and self-reported data, as well as the use of arbitrary cut-off points in analyses and some responder bias due to response rates. There was some loss to follow up in two surveys incorporated in the analysis sample. Regarding TFU2, of the initial cohort of 4056, 8.4% (n = 341) were unable to take part due to death, illness or incapacity or loss, and a further 17.7% (n = 719) withdrew from the cohort study, were unable to be contacted or declined to take part. Regarding Stage 3, the corresponding figures for loss to follow up were 8.5% (n = 346) and 839 (20.7%). An examination was undertaken of the representativeness of cohort participants compared to Australian Bureau of Statistics Estimated Residential Population age and sex data, and to demographic and risk factor information from a statewide health and wellbeing surveillance telephone survey (South Australian Monitoring and Surveillance System). It showed that by Stage 3, NWAHS had a higher proportion of females and older people, and that study participants were more likely to be employed, have a certificate or trade level of education, and to have a higher level of gross annual household income. They were also more likely to report better overall health, to be ex- or non-smokers and to be obese (based on self-report) [40].

Parental obesity has been suggested as one factor in a complex interaction between human behaviour, genetic disposition and the environment which can contribute to obesity. Ideally biomedical measures of the participants’ parents would be used, however the focus of our cohort study is the epidemiology of chronic disease and health-related risk factors among participants. Only limited information has been collected about participants’ parents, including their midlife body shape, occupation for most of their life and country of birth for initial exploration of life-course factors. Pictograms were originally formulated to determine the body build of the parents of both adoptees and biological parents where reported and/or measured information was not available, for example when parents have died [9], and were considered to be accurate representations [10]. These pictograms were also used in the Danish Nurse Cohort Study to determine familial predisposition to obesity [41]. Sorensen et al argue that while reports of body weight are less accurate than measurements, they are also less costly and enable epidemiological studies of obesity to be undertaken. They further highlight their value in separating extremes of the distribution, as well as allowing associations between relative weights of people to be investigated, particularly where absolute values are not available. In their study, participants were asked in 1979 to recall parental body shape during the early 1960s, some 15 or so years earlier, which was deemed to be sufficiently accurate [10]. This is similar to the approximate 17 year recall period asked of our study participants, whose mean age at Stage 3 was 57.6 years. Body shape at age 40 allows for consistency of recall across study participants, while avoiding earlier ages when parents are predominantly growing their families, as well as later middle age when people’s metabolism slows and weight gain is often experienced. It is also argued that while midlife parental height may be reported quite accurately, midlife parental weight would be less easily recounted. There have been some criticisms of the use of pictograms as representations of body shape, relating to coarseness of the scale with loss of information through the need to reduce the response to fit one of the options. Secondly, the restriction of the range of responses and the limited number of options available may lead to an inability to provide a standard deviation around the response. In addition, concern has been expressed regarding the method of presentation such as silhouettes being presented in ascending or descending order in one figure, rather than randomly presented as separate figures. There is also criticism regarding the scale of measurement in that silhouettes are inconsistent in size across the scale and all figures are the same height [42]. However, a number of studies have regarded pictograms to be a valid measure for the discrimination of overweight or obese compared to normal individuals, which can be reliably used for the estimation of BMI [8, 43].

The use of quick and easy to use pictograms to highlight a person’s risk of becoming obese like their parents may assist general practitioners with obesity management of their patients. A recent study reported that national guidelines regarding the documentation of height, weight and waist circumference were only being partially met, with 22.2% of patients having a recorded BMI score and 3.4% having a recorded waist circumference in their medical record [44]. Incorporating these measures may assist with improved health outcomes for people at risk of developing obesity-related diseases such as diabetes and hypertension.

## Conclusion

In conclusion, this is the first study, to our knowledge, to examine the influence of parental and adult offspring body shape in an Australian population. It provides further evidence that parental obesity increases the risk of obesity for adult offspring, both for overall body shape (as measured by BMI) as well as central adiposity (as measured by WC, WHR and WHtR). It also highlights the differences across four weight measures; two of which (BMI and WC) are used routinely to provide an indication of a person’s weight status, while providing evidence of the usefulness of another two measures (WHR and WHtR) in estimating the risk status regarding CVD and related factors such as hypertension. Using the adage “like mother, like daughter”(and similarly, father and son), pictograms could be used as a screening tool among young and early middle-aged adults in primary care settings to promote discussion regarding possible future risk of obesity, who may not recognise that this may be a problem in their family and for them in particular. This may lead to lifestyle changes to reduce weight, which may impact on the health-related consequences of obesity, particularly cardiometabolic disease.

## Supporting Information

S1 Table Comparison of demographic variables for baseline and Analysis Sample for adult sons and daughters (unweighted) (PDF)
