# Supplementary material for: Parental Midlife Body Shape and Association with Multiple Adult Offspring Obesity Measures: North West Adelaide Health Study
Source: PLoS One. 2015 Sep 10;10(9):e0137534. doi: 10.1371/journal.pone.0137534 (PMC4565704; doi:10.1371/journal.pone.0137534)
Supplement: S1 Table — (PDF) [file pone.0137534.s001.pdf]

S1 Table: Comparison of baseline demographic variables for those non-eligible or eligible in the Analysis Sample for adult sons and daughters (unweighted)

DEMOGRAPHICS (self-reported)	DAUGHTERS BASELINE Overall n	%	ANALYSIS SAMPLE Non-eligible n	%	*Eligible* n	%	p	SONS BASELINE Overall n	%	ANALYSIS SAMPLE Non-eligible n	%	*Eligible* n	%	p
**Age**														
18 to 29 years	228	10.7	144	14.4	*84*	***7.5***	<0.001	238	12.3	151	16.2	*87*	***8.7***	<0.001
30 to 39 years	370	17.4	187	18.7	*183*	***16.3***		306	15.8	142	15.3	*164*	***16.4***	
40 to 49 years	484	22.8	184	18.4	*300*	***26.6***		391	20.2	152	16.3	*239*	***23.9***	
50 to 59 years	415	19.5	140	14.0	*275*	***24.4***		380	19.7	131	14.1	*249*	***24.9***	
60 to 69 years	318	15.0	132	13.2	*186*	***16.5***		304	15.7	139	14.9	*165*	***16.5***	
70 years and over	309	14.5	211	21.1	*98*	***8.7***		313	16.2	215	23.1	*98*	***9.8***	
**Marital status**														
Married/defacto	1253	59.0	516	51.7	*737*	***65.5***	<0.001	1208	62.5	523	56.2	*685*	***68.4***	<0.001
Separated/ divorced	317	14.9	159	15.9	*158*	***14.0***		262	13.6	127	13.7	*135*	***13.5***	
Widowed	265	12.5	149	14.9	*116*	***10.3***		110	5.7	70	7.5	*40*	***4.0***	
Never married	274	12.9	164	16.4	*110*	***9.8***		344	17.8	204	21.9	*140*	***14.0***	
**Work status**														
Full time employed	489	23.0	195	19.5	*294*	***26.1***	<0.001	942	48.8	343	36.9	*599*	***59.8***	<0.001
Part time/casual employed	517	24.3	210	21.0	*307*	***27.3***		173	9.0	90	9.7	*83*	***8.3***	
Unemployed	55	2.6	31	3.1	*24*	***2.1***		91	4.7	62	6.7	*29*	***2.9***	
Home duties	538	25.3	274	27.5	*264*	***23.4***		17	0.9	9	1.0	*8*	***0.8***	
Retired	395	18.6	215	21.5	*180*	***16.0***		570	29.5	342	36.8	*228*	***22.8***	
Student	51	2.4	33	3.3	*18*	***1.6***		51	2.6	29	3.1	*22*	***2.2***	
Other	52	2.4	27	2.7	*25*	***2.2***		72	3.7	43	4.6	*29*	***2.9***	
**Highest educational qualification obtained**														
Up to secondary	1107	52.1	552	55.3	*555*	***49.3***	<0.001	642	33.2	354	38.1	*288*	***28.7***	<0.001
Trade/apprenticeship	83	3.9	45	4.5	*38*	***3.4***		601	31.1	283	30.4	*318*	***31.7***	
Certificate/diploma	586	27.6	254	25.5	*332*	***29.5***		416	21.5	176	18.9	*240*	***24.0***	
Bachelor degree or higher	263	12.4	107	10.7	*156*	***13.9***		210	10.9	76	8.2	*134*	***13.4***	
Other	11	0.5	4	0.4	*7*	***0.6***		11	0.6	3	0.3	*8*	***0.8***	
**Gross annual household income**														
Up to $12,000	363	17.1	216	21.6	*147*	***13.1***	<0.001	214	11.1	156	16.8	*58*	***5.8***	<0.001
$12,001 to $20,000	333	15.7	159	15.9	*174*	***15.5***		283	14.6	179	19.2	*104*	***10.4***	
$20,001 to $40,000	498	23.4	235	23.5	*263*	***23.4***		531	27.5	236	25.4	*295*	***29.4***	
$40,001 to $60,000	383	18.0	132	13.2	*251*	***22.3***		416	21.5	164	17.6	*252*	***25.1***	
$60,001 to $80,000	223	10.5	92	9.2	*131*	***11.6***		185	9.6	65	7.0	*120*	***12.0***	
More than $80,000	193	9.1	82	8.2	*111*	***9.9***		205	10.6	64	6.9	*141*	***14.1***	
**Total**	998	100.0	998	100.0	*1126*	***100.0***		1932	100.0	930	100.0	*1002*	***100.0***	

Note: χ^2 test undertaken between non-eligible and eligible participants in the analysis sample. "Not stated" not shown.
